# Diagnosis of Onychomycosis: From Conventional Techniques and Dermoscopy to Artificial Intelligence

**DOI:** 10.3389/fmed.2021.637216

**Published:** 2021-04-15

**Authors:** Sophie Soyeon Lim, Jungyoon Ohn, Je-Ho Mun

**Affiliations:** ^1^Alfred Health, Melbourne, VIC, Australia; ^2^Department of Dermatology, Seoul National University College of Medicine, Seoul, South Korea; ^3^Institute of Human-Environment Interface Biology, Seoul National University, Seoul, South Korea

**Keywords:** diagnosis, diagnostic imaging, onychomycosis, fungi, pathology, dermoscopy, reflectance confocal microscopy, artificial intelligence

## Abstract

Onychomycosis is a common fungal nail infection. Accurate diagnosis is critical as onychomycosis is transmissible between humans and impacts patients' quality of life. Combining clinical examination with mycological testing ensures accurate diagnosis. Conventional diagnostic techniques, including potassium hydroxide testing, fungal culture and histopathology of nail clippings, detect fungal species within nails. New diagnostic tools have been developed recently which either improve detection of onychomycosis clinically, including dermoscopy, reflectance confocal microscopy and artificial intelligence, or mycologically, such as molecular assays. Dermoscopy is cost-effective and non-invasive, allowing clinicians to discern microscopic features of onychomycosis and fungal melanonychia. Reflectance confocal microscopy enables clinicians to observe bright filamentous septate hyphae at near histologic resolution by the bedside. Artificial intelligence may prompt patients to seek further assessment for nails that are suspicious for onychomycosis. This review evaluates the current landscape of diagnostic techniques for onychomycosis.

## Introduction

Onychomycosis is a fungal nail infection caused by dermatophytes (60–70%), non-dermatophyte molds (NDMs) (20%) and yeast (10–20%) (1–3). It is the most common nail disorder encountered in clinical practice worldwide (4–6). It is a significant public health issue, as human to human transmission occurs *via* direct or indirect contact of surfaces contaminated with scales or keratin from infected patients. Risk of developing onychomycosis increases with advancing age. Thus, onychomycosis is likely to become an even more pertinent issue given the aging population (7, 8). Its other risk factors include diabetes, obesity, trauma, history of tinea pedis, and immunosuppression (9, 10). Onychomycosis is detrimental to patients' quality of life, as its physical appearance can cause significant psychological distress, and the localized pain in severely dystrophic nails can impede everyday living (11).

Onychomycosis is important to diagnose as it is curable with antifungal agents such as oral terbinafine, itraconazole, albaconazole, posaconazole, and fluconazole (12, 13). Topical antifungal solutions including ciclopirox 8%, amorolfine 5%, efinaconazole 10%, and tavaborole 5%, are used as adjuncts to oral agents in severe cases or as alternatives when oral agents are contraindicated or in mild cases (12). Due to costs and long treatment course lasting at least 3 months, patients often find difficulty adhering to therapy (12, 14–16). Ensuring that patients who do not have onychomycosis do not receive antifungal treatment is also important because oral antifungals have adverse systemic effects, including gastrointestinal disturbance and hepatotoxicity (13).

To diagnose onychomycosis, clinical suspicion needs to be confirmed with mycologic testing. This review highlights key characteristics of conventional diagnostic tools, including potassium hydroxide (KOH) testing, fungal culture and histopathology of nail clippings, and newly developed techniques, including dermoscopy, reflectance confocal microscopy, molecular assays and artificial intelligence.

## Clinical Features and Differential Diagnoses

**Figure 1:**
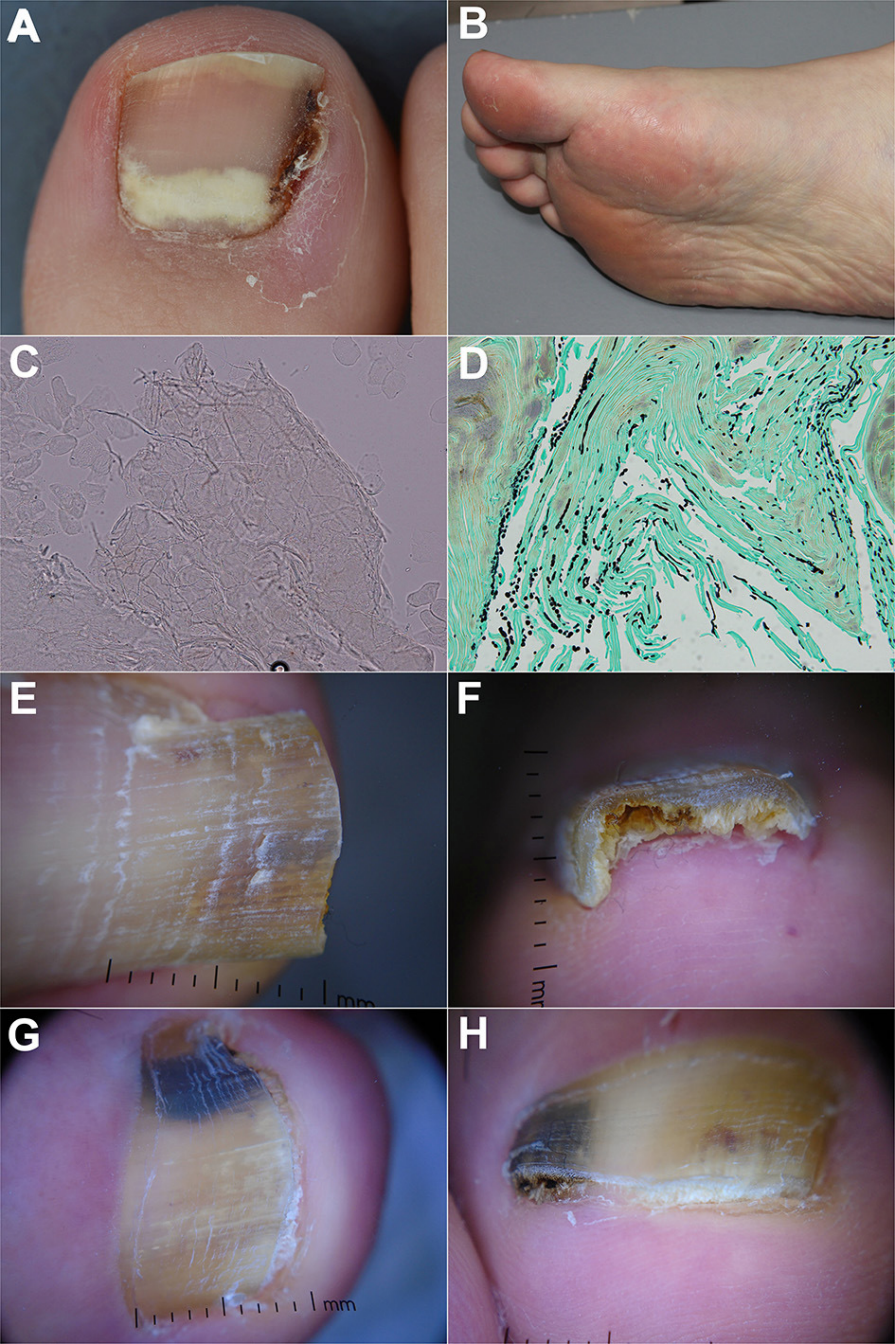
**(A)** Proximal subungual onychomycosis in a patient with systemic lupus erythematosus. **(B)** Abnormal plantar desquamation increases the likelihood of clinically diagnosing onychomycosis. Thus, the sole should also be examined while assessing onychomycosis. **(C)** KOH-test highlighting presence of fungal hyphae (×200 magnification). **(D)** Histopathology (nail clipping) with GMS staining showing numerous fungal hyphae in the nail plate (×400). The fungi are highlighted in black with GMS staining. **(E,F)** Dermoscopic examination of onychomycosis showing yellowish discoloration with spikes pattern and surface scales. Distal edge dermoscopy demonstrating subungual hyperkeratosis. **(G,H)** Dermoscopic examination of fungal melanonychia showing reverse triangular pattern, yellow streaks, black and yellow coloration, scales, and subungual hyperkeratosis.

Onychomycosis is typically characterized by a yellow or brown, brittle nail plate with subungual hyperkeratosis causing onycholysis (17–19). It is classified according to the site and involvement of disease: distal and lateral subungual, superficial, endonyx, proximal subungual, and total dystrophic onychomycosis (20). Distal and lateral subungual onychomycosis is the most common, thus the commonly described features of onychomycosis are those observed in this subtype (19, 20). Superficial onychomycosis presents with nail plate discoloration, superficial patches and transverse striae, and endonyx onychomycosis presents with lamellar splitting, discoloration and indentations (19, 20). Proximal subungual onychomycosis predominantly presents with a whitish area in the proximal nail plate (19, 20). This subtype is often seen in immunosuppressed patients, such as those with the human immunodeficiency virus, systemic lupus erythematosus or on immunosuppressants (Figure 1A) (10).

According to a prospective cross-sectional diagnosis study in seven dermatology outpatient clinics, features that increase the likelihood of clinically diagnosing onychomycosis are previous diagnosis of fungal disease in feet [likelihood ratio (LR+) for a positive result = 1.84], abnormal plantar desquamation involving > 25% of the sole (LR+ = 3.61), interdigital tinea pedis (LR+ = 1.46) and onychomycosis being considered the most probable diagnosis by dermatologists (LR+ = 1.46) (9). When abnormal plantar desquamation is coupled with onychomycosis being considered the most likely diagnosis, probability of diagnosing onychomycosis is 81% (Figure 1B). Onychomycosis can present alongside green nail syndrome. A retrospective analysis of green nail syndrome patients at referral centers revealed fungal co-infection in 65.2% of patients (21). Onychomycosis can present with a dermatophytoma, a compact ball of fungal filaments and large spores which appears as a white/yellow or orange/brown longitudinal streak in the nail plate (22, 23). Features of severe or long-standing disease include dermatophytoma, worsening subungual hyperkeratosis, expanding area of disease involvement and closer proximity of disease to the nail matrix (24). Severe disease can cause pain secondary to onychocryptosis (ingrown nail), nail bed infection and partial or complete nail plate loss (18).

### Differential Diagnoses

#### Trachyonychia

Trachyonychia is an inflammatory nail disease which is commonly idiopathic but can be secondary to systemic inflammatory conditions such as alopecia areata and nail psoriasis (25, 26). It is characterized by a rough and brittle nail plate (opaque trachyonychia) or, less frequently, an opalescent nail plate lined with small geometric pits (shiny trachyonychia) (27). Trachyonychia is diagnosed clinically and dermoscopy is a useful diagnostic aid. Key dermoscopic features of trachyonychia that differentiate it from onychomycosis include red coloration, longitudinal ridging, involvement of the proximal nail plate (at >50% of the nail plate width), splinter hemorrhages, pitting, and onychoschizia (nail plate splitting) (28).

#### Onycholysis

Onycholysis involves separation of the nail plate from the nail bed due to repetitive trauma, phototoxicity, contact dermatitis, underlying tumor or infection, nail psoriasis, or nail lichen planus (29). Onycholysis secondary to trauma can be differentiated from that resulting from onychomycosis on dermoscopic examination. The proximal border of onycholysis is linear with trauma, but jagged with spiked edges in onychomycosis (30).

#### Onychomadesis

Onychomadesis is characterized by complete separation and shedding of the nail plate from the proximal nail bed due to temporary nail matrix arrest (31, 32). Conditions that cause nail matrix arrest include infections such as hand-foot-and-mouth disease and varicella, trauma, chemotherapy, anticonvulsants, lichen planus, and Kawasaki disease (32). Nails affected by onychomadesis are at risk of developing onychomycosis (33).

#### Onychocryptosis

Onychocryptosis (ingrowing nail) involves the nail plate burying within the periungual skin, causing painful inflammation, infection and granulation (34). Although it is observed secondary to severe onychomycosis, it is primarily associated with genetic disposition, trauma, poorly-fitted shoes, and incorrect nail trimming (31, 34).

#### Nail Squamous Cell Carcinoma

Nail SCC is the most common malignant nail disorder and can clinically resemble onychomycosis (35, 36). Like onychomycosis, immunosuppression increases the risk of developing nail SCC. Other risk factors include chronic sun exposure, human papillomavirus infection and trauma (37). Diagnosis delays occur frequently as nail SCC presents with non-specific clinical features, including subungual tumor, lateral onycholysis, subungual hyperkeratosis, painless nail bed erosion, ulceration, purulent discharge, serous ooze, bleeding and nail loss (37, 38). Therefore, nail dystrophies refractory to treatment need to be carefully investigated.

#### Nail Apparatus Melanoma

Nail apparatus melanoma is another malignant nail disorder that warrants prompt diagnosis (39). Its prognosis is poorer than other melanomas due to diagnosis delays resulting from non-specific clinical features and high incidence of amelanosis (40, 41). Nail apparatus melanoma commonly presents with a ≥3 mm-thick brown or black band with variegated borders, nail dystrophy and Hutchinson's sign, where the proximal and/or lateral nail fold is pigmented (41–44). Nail apparatus melanoma can be mistaken for onychomycosis and they may be co-exist with onychomycosis, thus detailed investigation is warranted in onychomycosis with malignant features (45, 46).

## Conventional Diagnostic Tools

To diagnose onychomycosis, confirmatory mycologic testing is essential. The potential harm caused by misdiagnosis and inappropriate use of empiric antifungal therapy outweigh the cost benefit of bypassing mycologic testing (18, 47, 48).

### Potassium Hydroxide Testing

Direct potassium hydroxide (KOH) testing is a simple, quick and inexpensive technique integral to dermatological practice for identifying fungal organisms (Figure 1C) (49, 50). It involves retrieving the specimen from the nail bed and underneath the nail plate then dissolving it in KOH (51). KOH dissolves the keratin, allowing microscopic visualization of the fungal septate hyphae (51). Specimens can be further treated with stains such as Calcofluor White, Evans Blue, Gram, Giemsa, and India ink (52, 53).

KOH testing has 61% sensitivity and 95% specificity (51). It is cost-effective and can determine the presence of fungal organisms within an hour. However, it cannot specify the exact type of pathogenic organism (19). Retrieving adequate amount of specimen is critical in ensuring the success of KOH testing. To optimize accuracy, specimens should not be interpreted immediately after applying KOH, as it takes at least 15–30 min for the KOH to adequately dissolve the keratin (19). Overall, KOH testing is a quick and inexpensive diagnostic tool for confirming presence of fungi in nails, enabling clinicians to commence treatment for onychomycosis (Table 1).

**Table 1:** Summary of diagnostic techniques for onychomycosis.

	**Sensitivity**	**Specificity**	**Advantages**	**Disadvantages**
KOH testing (51)	61% (44–100%)	95% (75–100%)	Easy to conduct	Diagnostic accuracy dependent on examiner's expertise
			Inexpensive	Cannot identify pathogen subtype
			Quick results (15–60 min)	
Fungal culture (51)	56% (29–82%)	99% (83–100%)	Can identify pathogen subtype	Low sensitivity
				Delay in results (up to 1 month)
Histopathology (51)	84% (61–93%)	89% (44–100%)	Most sensitive conventional mycological test	Expensive
				Cannot specify pathogen subtypes
Nail dermoscopy	Jagged onycholytic edge with spikes: 86.4% (54), 100% (30)	Jagged onycholytic edge with spikes: 58.3% (54), 100% (30)	Bedside tool, non-invasive	Cannot demonstrate presence of fungi
	Longitudinal striae: 25% (54), 86.5% (30)	Longitudinal striae: 83.3% (54), 100% (30)	Quick results	
	Ruins aspect: 59.1% (54)	Ruins aspect: 91.7% (54)	Inexpensive	
	Homogenous opacity: 34.1% (54)	Homogenous opacity: 83.3% (54)		
Reflectance confocal microscopy	52.9% (55), 79.5% (56), 91.67% (57)	57.58% (57), 81% (56), 90.2% (55)	Bedside tool, non-invasive	Moderate sensitivity and specificity
				Cannot assess thick nails
Polymerase chain reaction	85% (58)	94% (58)	Can identify pathogen subtype	Assays still under development
	87.3% (59)	94.3% (59)	High sensitivity	Risk of false positives
	100% (60)	100% (60)	Can deliver results with small amount of sample	
	Pandermatophyte assay: 90% (61)	Pandermatophyte and panfungal assays: NR		
	Panfungal assay: 47% (61)			
Flow cytometry and mass spectrometry	NR	NR	Can theoretically identify pathogen subtype	Experimental
Artificial intelligence	70.2% (62)	72.7% (62)	Inexpensive	Still under development: require improving dataset and considering method of distribution to clinicians
	82.7–96% (63)	69.3–96.7% (63)	Can be used by patients to screen for highly suspicious nails	Not confirmatory technique

*NR, not reported*.

### Fungal Culture

Fungal culture can identify the specific pathogen subtype (53, 64). After removing debris from the nail plate, the subungual specimen is cultured in Sabouraoud dextrose agar (SDA) at 26–30°C for up to a month (18, 52). The media is treated with chloramphenicol and gentamicin to prevent bacteria from interfering with fungal growth (52). Laboratories often culture the subungual specimen in SDA with and without cycloheximide which prevents growth of NDMs. Repeat cultures are required to diagnose NDM onychomycosis, as NDMs are common contaminants of skin surfaces (18).

Fungal culture has 99% specificity, its pooled sensitivity is 56% (range 29–82%) according to a recent meta-analysis (51, 53). Sensitivity of fungal culture is largely dependent on the expertise of the testing center (65). Although specialized mycology centers may report fewer false negatives, most tertiary centers and general clinics observe low sensitivity rates. Clinical utility of fungal culture is further limited by delays in retrieving results (several weeks to months). Therefore, fungal culture is recommended when identifying the fungal organism is necessary (53).

### Histopathology (Nail Clipping)

Histopathological assessment involves examining the microscopic features of nail clipping specimens embedded in paraffin blocks (18, 52). To acquire an adequate nail sample for examination, at least 4 mm of the free edge of the nail plate should be retrieved using a dual-action or heavy-duty nail nipper (66). Samples can be transported to the laboratory in a dry container or in formaldehyde (67). Softening nail samples before routine processing with solutions such as chitin-softening agent, 4% phenol or 10% Tween 40, facilitates sectioning and thus optimizes the quality of sections (66, 67). After paraffin embedding and sectioning, stains highlight presence of fungi (Figure 1D). Using the periodic acid-Schiff (PAS) staining method, periodic acid oxidizes hydroxyl groups of the cell wall in spores, hyphae, pseudohyphae and yeasts into aldehydes (52, 53). These then react with Schiff to produce a red color (52, 53). Using the Grocott-Gomori methanamine silver (GMS) staining method, the chromic acid oxidizes the cell wall then reduces the methanamine silver nitrate into metallic silver to produce a dark brown color (53).

Histopathology of nail clippings is highly sensitive (84%) and specific (89%) (51). It can also be stored and retrospectively reviewed in refractory cases, as the paraffin embedding allows for long-term storage. Histopathology is however rather costly than KOH testing and unable to specify the exact subtype or viability of the causative organism (18, 19, 53, 68).

## New Diagnostic Tools

### Nail Dermoscopy (Onychoscopy)

Nail dermoscopy (onychoscopy) is a non-invasive bedside tool that allows clinicians to visualize microscopic features of abnormal nails. Key dermoscopic features of distal and lateral subungual onychomycosis include a jagged proximal edge of the onycholytic area with spikes and longitudinal striae (Figures 1E,F) (30, 54). These features and a ruins aspect are associated with total dystrophic onychomycosis (54). Homogenous opacity is found in superficial onychomycosis (54).

Onychomycosis can also present with longitudinal melanonychia (fungal melanonychia). In such cases, white or yellow streaks, non-longitudinal homogenous pattern, yellow coloration, reverse triangular pattern, subungual hyperkeratosis, multicolor pattern and nail scaling are positive predictors of fungal melanonychia (Figures 1G,H) compared to nail matrix naevi or subungual melanomas (69). As nail dermoscopy is quick, non-invasive and inexpensive, it has the potential to help physicians identify onychomycosis by the bedside and decide whether to proceed to mycological assessment (70).

### Reflectance Confocal Microscopy

Reflectance confocal microscopy (RCM) is a real-time imaging tool that allows clinicians to observe features of abnormal nails at near-histologic resolution by the bedside. It uses a 830 nm laser in reflectance mode which divides the nail unit into thin horizontal sections for examination (56). Reflectance confocal microscopy of onychomycosis reveals networks of bright filamentous septate hyphae (56, 71, 72). It has 52.9–91.67% sensitivity and 57.58–90.2% specificity for detecting onychomycosis (55–57). Reflectance confocal microscopy is expensive and not subsidized in many countries, and further studies are needed to support its utility in the clinical setting (72). Therefore, it is yet to be integrated into clinical practice in many countries. In addition, it is difficult to assess thick nails with RCM as its depth of imaging is limited to ~200 μm.

### Molecular Assays

Molecular assays including polymerase chain reactions (PCR), flow cytometry and mass spectrometry are advanced diagnostic tools that involve analysis of the fungal DNA causing onychomycosis.

Polymerase chain reactions involves amplifying the fungal DNA then detecting this with specialized fluorescent primers (73). Therefore, it can detect small amounts of pathogenic organisms within nails (74). Real-time PCR is the most frequently used form of PCR as it is relatively simple to conduct, can detect multiple organisms and has a low risk of contamination (73, 74). Various assays have been developed to facilitate commercial use of PCR technology when detecting dermatophytes, but these are not widely available (74). They report sensitivity of 85–100% and specificity of 94–100% (58–60).

Hafirassou et al. investigated usefulness of panfungal and pandermatophyte assays for real-time PCR compared to fungal culture in detecting onychomycosis (61). The pandermatophyte assay was 90% sensitive relative to culture. The panfungal assay showed a low sensitivity of 47% relative to culture due to multiple fungal species residing within diseased and healthy nails, as demonstrated by the candida and aspergillus assays. Further studies are warranted to examine PCR use in a real-world setting and reduce the risk of false positives.

Other molecular assay techniques include flow cytometry and mass spectrometry. Flow cytometry separates cells according to size, granulosity and presence of DNA and protein markers (18). Mass spectrometry involves charging chemical species and separating ions according to their mass-to-charge ratio (18, 53, 75). These techniques are experimental, requiring further research and development prior to integration into clinical practice.

### Artificial Intelligence

Artificial intelligence (AI) has caused a recent paradigm shift in clinical medicine (76, 77). Its diagnostic performance has been shown to be comparable to that of specialist clinicians in identifying diabetic retinopathy and skin cancer (78, 79). Many dermatologists recognize that AI has great potential to improve dermatologic care (80, 81). Currently, it is mainly explored in the setting of skin cancer, ulcers, psoriasis and other inflammatory skin diseases, predicting skin-sensitizing substances, histopathological assessment, and gene expression profiling (82).

Developing a large database of photographs capturing a wide range of disease presentations is critical in AI training (82). Onychomycosis is an ideal candidate for AI as it is a common condition with minimal racial differences (63). Clinics worldwide can contribute to the database, and the AI technology would not be limited to specific populations.

In 2018, Han et al. developed AI for diagnosing onychomycosis (63). Two datasets, A1 (*n* = 49,567) and A2 (*n* = 3,741), were generated. A2 consisted of images of clinically diagnosed onychomycosis (63). For A1, standardized clinical images were generated by a hand and foot image selecting convolutional neural network (CNN), followed by a nail part extracting regional CNN (R-CNN) then a fine image CNN. Two CNN algorithms, ResNet-152 and VGG-19, were then trained to classify nails as onychomycosis or one their differential diagnoses using the two datasets. These algorithms were then validated against datasets (B1, B2, C, and D) of mycologically confirmed onychomycosis cases.

Algorithms trained with A1 were more accurate in diagnosing onychomycosis than those trained with A2. Moreover, the AI (two-layered feedforward neural networks computing the combined output of ResNet-152 and VGG-19) achieved test sensitivity/specificity/area under the curve values of (96.0/94.7/0.98), (82.7/96.7/0.95), (92.3/79.3/0.93), and (87.7/69.3/0.82) for the B1, B2, C, and D datasets, respectively. AI performed better than most of the 42 dermatologists. The Youden index (sensitivity + specificity−1) of AI which reflects its diagnostic accuracy was significantly higher than that of the dermatologists (*p* = 0.01) when evaluating the B1 and C datasets.

More recently, the group reported that the deep neural network at operating point achieved 70.2% sensitivity, 72.7% specificity and AUC of 0.75 in diagnosing onychomycosis in a prospective cohort of 90 patients (62). This was comparable to the performance of dermoscopy (sensitivity 72.7%, specificity 72.9%, AUC 0.755; *p* = 0.952) and experienced dermatologists (mean Youden index 0.230 ± 0.176; *p* = 0.667).

Although there is limited literature on this topic, these results showed that AI has the potential to assist clinicians decide whether they should test nails for onychomycosis. AI can also help improve telemedicine, as it can act as an accessible resource for patients to evaluate their own nails and, if needed, promptly initiate a formal clinician review. To further develop AI, the database should be optimized to include rigorously confirmed onychomycosis and various non-onychomycosis onychopathies that are evaluated with relevant mycological examinations. Finding a suitable means of distributing this technology to the public is also necessary. Finally, whilst integrating AI into clinical practice is important, dermatologists should use their clinical judgment to prevent overdiagnosis and excessive testing which would increase the burden on health care costs.

## Conclusion

**Figure 2:**
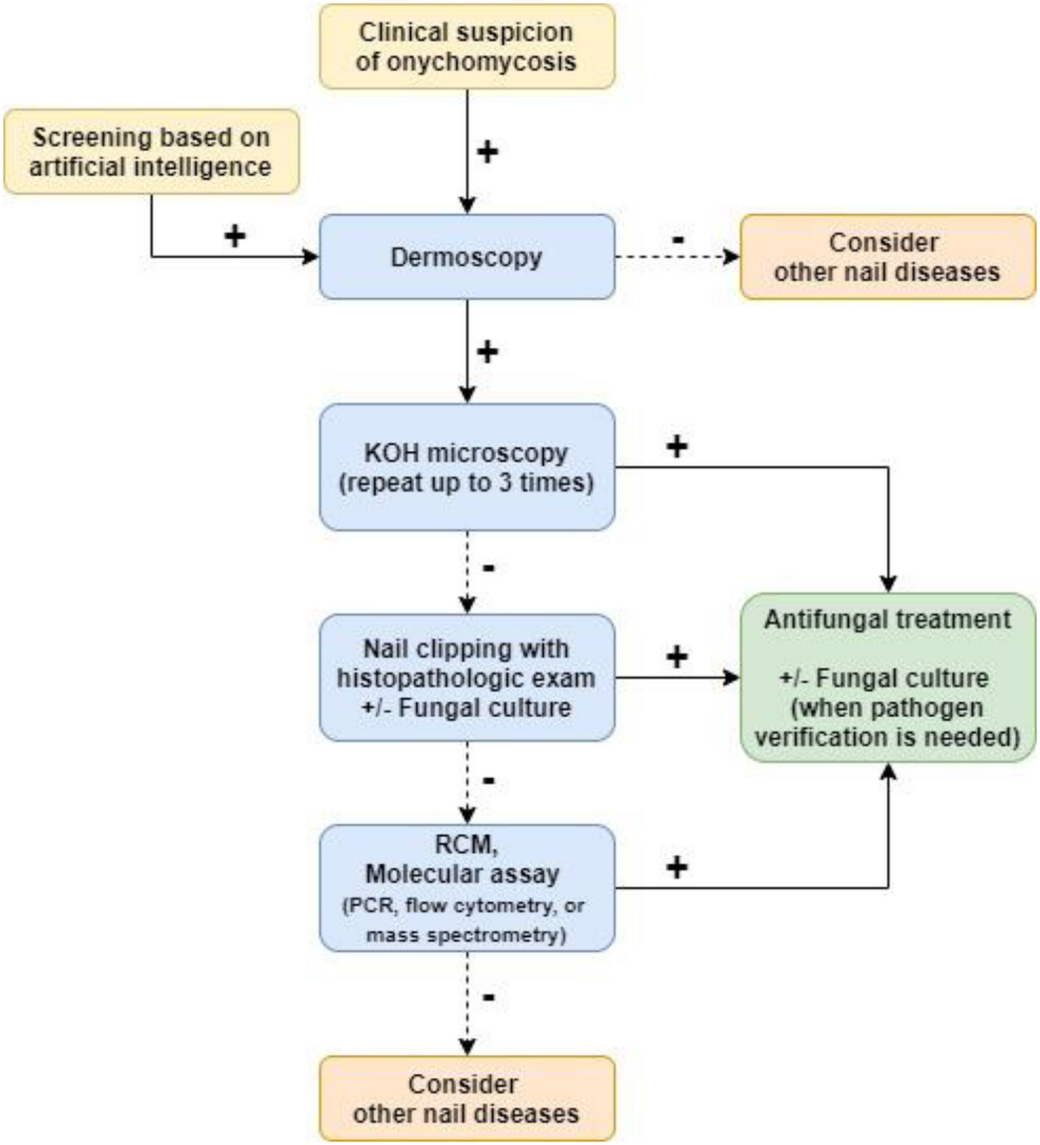
A flowchart for diagnosing onychomycosis.

A wide range of conventional and newly developed tools help diagnose onychomycosis. Each tool has its advantages and disadvantages, and combining these tools improves the sensitivity and specificity of testing (51). KOH testing is best for prompt initial diagnosis. In ambiguous cases, histopathologic assessment of affected nail plates can identify presence of fungi. Fungal culture can be used when the pathogen subtype needs to be specified. Dermoscopy can assist clinicians identify nails that are highly suspicious for onychomycosis. Reflectance confocal microscopy allows visualization of hyphae by the bedside but is not widely available, and molecular assays may act as supplementary diagnostic tests but require more research. AI has the potential to help patients identify affected nails and seek further medical assessment. A diagnostic algorithm integrating these tools can help maximize clinicians' accuracy of diagnosing onychomycosis (Figure 2).

## Author Contributions

SL: data acquisition, analysis, and manuscript drafting. JO: manuscript critical revision. J-HM: conception of work, manuscript critical revision, and final approval. All authors: contributed to the article and approved the submitted version.

## Conflict of Interest

The authors declare that the research was conducted in the absence of any commercial or financial relationships that could be construed as a potential conflict of interest.

## Abbreviations

AI: artificial intelligence
AUC: area under curve
GMS: Grocott-Gomori methanamine silver
KOH: potassium hydroxide
LR+: likelihood ratio for a positive result
NDM: non-dermatophyte mold
PAS: periodic acid-Schiff
PCR: polymerase chain reaction
RCM: reflectance confocal microscopy
R-CNN: region-based convolutional neural network
SCC: squamous cell carcinoma
SDA: Sabouraoud dextrose agar.
